# Handgrip strength and balance in older adults following withdrawal from long-term use of temazepam, zopiclone or zolpidem as hypnotics

**DOI:** 10.1186/1471-2318-14-121

**Published:** 2014-11-21

**Authors:** Janne Nurminen, Juha Puustinen, Ritva Lähteenmäki, Tero Vahlberg, Alan Lyles, Markku Partinen, Ismo Räihä, Pertti J Neuvonen, Sirkka-Liisa Kivelä

**Affiliations:** Department of Family Medicine, University of Turku, Lemminkäisenkatu 1, FI-20014 Turku, Finland; Department of Neurology, Division of Neuroscience, Turku University Hospital, University of Turku, Turku, Finland; Satakunta Central Hospital, Pori, Finland; Medical Teaching and Research Health Centre, University Consortium of Pori, Pori, Finland; Department of Biostatistics, University of Turku, Turku, Finland; College of Public Affairs, University of Baltimore, Baltimore, Maryland USA; Härkätie Health Centre, Lieto, Finland; Division of Social Pharmacy, University of Helsinki, Helsinki, Finland; Department of Neurology, Vitalmed Research Center, Helsinki Sleep Clinic, University of Helsinki, Helsinki, Finland; Department of Clinical Pharmacology, University of Helsinki, Helsinki University Central Hospital, HUSLAB, Helsinki, Finland

**Keywords:** Benzodiazepines, Temazepam, Zopiclone, Zolpidem, Withdrawal, Handgrip strength, Balance, Improvement, Older adults

## Abstract

**Background:**

Benzodiazepines and related drugs affect physical functioning negatively and increase fall and fracture risk. As impaired muscle strength and balance are risk factors for falls, we examined the effects of hypnotic withdrawal on handgrip strength and balance in older adult outpatients during and after long-term use of temazepam, zopiclone and zolpidem (here collectively referred to as “benzodiazepines”).

**Methods:**

Eighty-nine chronic users (59 women, 30 men) of temazepam, zopiclone or zolpidem aged ≥55 years participated in a benzodiazepine withdrawal study. Individual physician-directed withdrawal was performed gradually over a one-month period and participants were followed up to six months. Handgrip strength was assessed using a handheld dynamometer, and balance using the Short Berg’s Balance Scale during the period of benzodiazepine use (baseline), and at 1, 2, 3 weeks, and 1, 2 and 6 months after initiating withdrawal. Withdrawal outcome and persistence were determined by plasma benzodiazepine-determinations at baseline and at four weeks (“short-term withdrawers”, n = 69; “short-term non-withdrawers”, n = 20), and by interviews at six months (“long-term withdrawers”, n = 34; “long-term non-withdrawers”, n = 55). Also most of the non-withdrawers markedly reduced their benzodiazepine use.

**Results:**

Within three weeks after initiating withdrawal, handgrip strength improved significantly (P ≤ 0.005) compared to baseline values. Among women, long-term withdrawers improved their handgrip strength both when compared to their baseline values (*P =* 0.001) or to non-withdrawers (*P* =0.004). In men, improvement of handgrip strength from baseline was not significantly better in withdrawers than in non-withdrawers. However, men did improve their handgrip strength values compared to baseline (*P =* 0.002). Compared to balance test results at baseline, withdrawers improved starting from the first week after withdrawal initiation. There was, however, only a borderline difference (*P =* 0.054) in balance improvement between the long-term withdrawers and long-term non-withdrawers. Of note, the non-withdrawers tended to improve their handgrip strength and balance compared to baseline values, in parallel with their reduced benzodiazepine use.

**Conclusions:**

Withdrawal from long-term use of benzodiazepines can rapidly improve muscle strength and balance. Our results encourage discontinuing benzodiazepine hypnotics, particularly in older women who are at a high risk of falling and sustaining fractures.

**Trial registration:**

EU Clinical Trials Register: EudraCT2008000679530. Registered 31 October 2008

## Background

Benzodiazepines and benzodiazepine related drugs (here collectively referred to as “benzodiazepines”) are widely used to treat insomnia and other conditions in older adults [1–3]. Their adverse effects include a decline in physical functioning [4] and an increased risk for falls and fractures [5–7]. Benzodiazepines are a major cause of hospitalization, permanent loss of physical function and even death [8]. Fall and fracture prevention studies show that withdrawal of psychotropic drugs decreases older adults’ risk of falling [9, 10].

Impaired balance and muscle strength are associated with falls, especially in older adults [11]. Benzodiazepines can affect balance and physical functioning negatively [12, 13]. However, most studies in this field have examined benzodiazepine-effects following a single dose, and longitudinal studies have yet to be performed [14]. One mechanism for the possible reduction of risk of falls and fractures after benzodiazepine withdrawal could be enhanced balance and muscle strength. Although the use of psychoactive medications has been connected to lowered muscle strength [15], clinical studies specifically concerning benzodiazepines and muscle strength are rare [16].

Only a few studies exist on the effect of withdrawal from long-term use of benzodiazepines on muscle strength and balance in older adults [17, 18]. These studies reported improved daily functions and balance in nursing home residents after benzodiazepine withdrawal. However, the study by Habraken et al. relied exclusively on observational data to assess the level of daily functioning during and after lorazepam use [17]. The study by Tsunoda et al. had a relatively small sample size and the population was rather selected as most of their subjects had schizophrenia or dementia [18]. Furthermore, no subjects in these two studies [17, 18] was using temazepam, zopiclone or zolpidem.

The aim of the current study was to assess whether withdrawal from long-term use of temazepam, zopiclone or zolpidem enhances handgrip strength and balance in older outpatients – a question that has not previously been the subject of rigorous research.

## Methods

### Study design and participants

This study is a part of the Satauni Study, a prospective benzodiazepine withdrawal study, details of which are reported elsewhere [19, 20]. The Study protocol was approved by the Ethics Committee of Satakunta Hospital District and by the National Agency for Medicines of Finland (EU Clinical Trials Register: EudraCT2008000679530). A written informed consent was received from each participant before they entered the trial in which the participant’s benzodiazepine dose was planned to be tapered off over a one month period.

**Figure 1:**
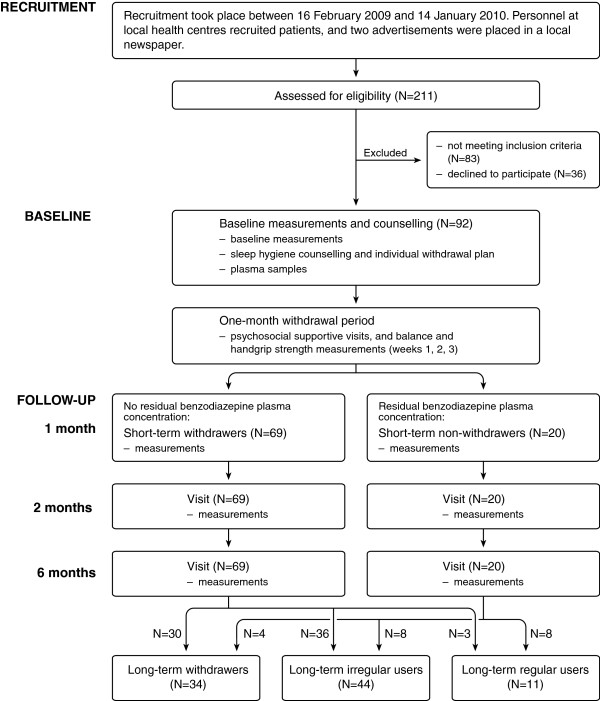
**Benzodiazepine withdrawal study flow chart.** Legend: There were two dropouts at week 3 and one at week 6.

Participants were primary health care outpatients living in the Province of Satakunta in western Finland [19]. Persons 55 years of age or over who had been using temazepam, zopiclone or zolpidem as hypnotics on a regular long-term basis (night-time use >1 month prior to enrollment) to treat primary insomnia were included in the study (Figure 1). A nurse performed the preliminary telephone or e-mail screenings, and a physician met the potential participants for screening, recruitment and to obtain informed consent [19]. The main exclusion criteria were use of antipsychotic or antiepileptic medication or benzodiazepines other than those identified above; a current or past history of alcohol or drug abuse; severe psychiatric, neurological or autoimmune disease; and smoking more than 10 cigarettes a day. Of the 211 subjects assessed for eligibility, 83 did not meet the inclusion criteria, 36 declined to participate and 3 withdrew from the study. Eighty nine outpatients (59 women and 30 men; mean age 67; range 55–91 years) met the criteria. Individual physician-directed withdrawal was performed gradually over a one-month period and participants were followed up to six months (Figure 1). Participants’ handgrip strength and balance test data are reported in the present study. In the original Satauni Study melatonin (2 mg) was compared double-blindly to placebo. It reported that patients neither benefited from melatonin use in benzodiazepine withdrawal nor did the melatonin cause adverse effects [19]. Based on that result, data from the both groups were pooled and analyzed together in this study.

### Measurement and classifications

At baseline (that is, before initiating withdrawal) data concerning health behavior, diseases, sleep behavior and medication use were collected and a thorough clinical examination was performed [19]. Venous blood samples were taken at baseline and one month after the start of withdrawal to determine the residual plasma concentration of benzodiazepines (procedure details are described elsewhere [19]). Participants with no plasma concentration of benzodiazepine at one month after the start of the withdrawal were classified as short-term withdrawers (n = 69), and those with a measurable concentration as short-term non-withdrawers (n = 20) (Figure 1). The division of participants into long-term withdrawers (n = 34) and long-term non-withdrawers (n = 55; including 44 irregular users and 11 daily users) was determined by interviewing the participants and checking their medical records and prescriptions in detail over the six months period after the start of withdrawal [19]. The demographic data at baseline for the short-term withdrawers and non-withdrawers as well as long-term withdrawers and non-withdrawers are given in Table 1. Most of the non-withdrawers also markedly (≥50 %) decreased their benzodiazepine use, and many of those were without benzodiazepines for several weeks or months, but they were classified as non-withdrawers if they were not abstinent up to the 6 month mark. At baseline, the short-term withdrawers and non-withdrawers did not differ by age, gender, marital status, education, body measures, self-reported quality of life, number of medications, duration of benzodiazepine use or alcohol consumption, as described elsewhere [19]. However, there were more smokers among the short-term non-withdrawers. The short-term non-withdrawers also had more depressive symptoms than did the short-term withdrawers.

**Table 1:** **Demographic data at baseline**

	Short-term withdrawers	Short-term non-withdrawers	Long-term withdrawers	Long-term non-withdrawers
	N = 69	N = 20	N = 34	N = 55
	(45 women, 24 men)	(14 women, 6 men)	(19 women, 15 men)	(40 women, 15 men)
Age, years (mean ± SD)	67 ± 7	67 ± 6	65 ± 7	68 ± 7
Number of medications (median [LQ, UQ])	4.0 [3.0, 5.0]	4.0 [3.0, 5.5]	4.0 [3.0, 6.0]	4.0 [3.0, 5.0]
	N	N	N	N
Duration of benzodiazepine use				
Less than 5 years	12	2	6	8
5 to 10 years	34	8	17	25
10 years or longer	23	10	11	22
Benzodiazepine use (daily dose range)				
Temazepam (10–30 mg)	9	4	3	10
Zopiclone (3.75-30 mg)	39	11	19	31
Zolpidem (5–20 mg)	21	5	12	14
Depression				
Not depressed (GDS-15 sum score <6)	63	16	32	47
Depressed (GDS-15 sum score ≥6)	5	4	2	7
Smoking				
Non-smoker	67	16	32	51
Smoker	2	4	2	4
Use of alcohol				
Non-user	16	1	5	12
Once a month or more seldom	25	6	13	18
2-4 times a month	17	9	9	17
2 times a week or more often	10	3	7	6
Self-reported health				
Good	17	1	9	9
Fair	44	14	18	40
Poor	8	5	7	6

N = number of participants.

GDS-15 = Geriatric Depression Scale (15 point version).

SD = Standard deviation.

LQ = Lower quartile.

UQ = Upper quartile.

### Handgrip strength and balance measurements

Handgrip strength and balance were measured during this prospective study at baseline (immediately before the withdrawal), and at 1, 2, 3, and 4 weeks after beginning the withdrawal, and at the 2 and 6 month follow-ups. Handgrip strength and balance measurements were performed by a trained nurse blinded to withdrawal success and persistence of benzodiazepine abstinence.

Handgrip strength (both hands three times consecutively) was measured with a Saehan (Jamar®) dynamometer in kilograms (kg) [21]. The average value of the stronger hand was recorded. Balance was assessed with Short Berg’s Balance Scale (BBS-9) [22], a 9-item version of the original BBS [23]. The BBS-9’s range is from 0 to 36 [22]. Data on the BBS-9 was divided into two groups, i.e. participants scoring less than 33 points and those scoring 33 points or more. There were two reasons why BBS-9 scores were not used as a continuous variable. First, scores below 33 are associated with increased fall risk [24], and second, distribution of BBS-9 scores was extremely negatively skewed.

### Statistical analyses

Participants who had used temazepam, zopiclone or zolpidem were analyzed together because the small numbers of users of the individual drugs did not allow adequate statistical power (Table 1). At baseline, differences between the withdrawers and non-withdrawers were tested with the Mann–Whitney U-test, two-sample t-test or χ^2^ -test.

As the mean handgrip strength (±SD) of men at baseline, 42.4 kg (6.2 kg), was significantly (*P <* 0.001) greater than that of women, 22.8 kg (5.2 kg), their data was analyzed separately. Handgrip strength between withdrawers and non-withdrawers was compared at baseline and during subsequent time points with repeated measures analysis of variance using a heterogeneous compound symmetry covariance structure. Group (short-term withdrawers/non-withdrawers; long-term withdrawers/non-withdrawers) was used as a fixed factor and time was used as a repeated factor. Interaction effects of group × time were also tested. Dunnett’s multiple comparison method was used in comparing different follow-up time points to baseline.

For the balance analyses, we pooled data from men and women, since there was no statistically significant difference in the BBS-9 data between the genders at baseline (*P =* 0.924). BBS-9 scores (≥33 vs. <33) were compared between withdrawers and non-withdrawers at baseline and during subsequent time points. Binary logistic regression analysis with generalized estimating equations (GEE) with an exchangeable working correlation matrix was used. Group was treated as a fixed factor and time was treated as a repeated factor in the logistic model. Interaction effects of group × time were also tested.

P-values less than 0.05 were considered as statistically significant. SAS version 9.2 and SAS Enterprise Guide 4.1 (SAS Institute Inc., Cary, NC, USA) were used in the statistical analyses.

## Results

### Demographic data at baseline and withdrawal results

Baseline data collected before initiating withdrawal is given in Table 1. In this table, the results are presented separately for short-term withdrawers and non-withdrawers as well as for long-term withdrawers and non-withdrawers. There were more smokers among short-term non-withdrawers than short-term withdrawers (*P =* 0.022). No other statistically significant differences were found for the variables presented in Table 1.

It is noteworthy that many of the non-withdrawers (both men and women) had also reduced their benzodiazepine use. At baseline, all participants were daily users of benzodiazepines. Six months after withdrawal initiation, 19 women and 15 men had completely stopped using benzodiazepines. Furthermore, 28 women and nine men were using benzodiazepines once a week or less frequently, and four women and three men were using a hypnotic 2–6 times a week, respectively. Eight women and three men were daily users of benzodiazepines at six months after beginning of the withdrawal.

### Handgrip strength in women

**Figure 2:**
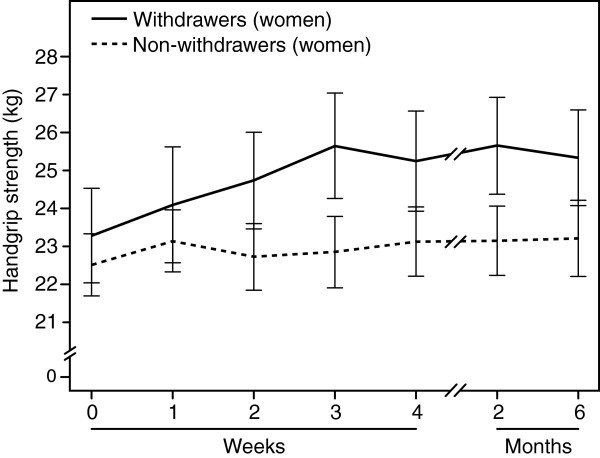
**Associations between medication withdrawal result and handgrip strength in women.** Legend: Mean (± standard error) of handgrip strength (kg) in women (*n =* 59) by success of long-term (“SIX MONTHS”) withdrawal at different time points (0 = before withdrawal, at 1, 2, 3 and 4 weeks after beginning of withdrawal, and at 2 and 6 months follow-up period). Women who were benzodiazepine-free at 6 months (n = 19; “long-term withdrawers”) improved their handgrip strength more than those who were not benzodiazepine-free at 6 months (n = 40; “non-withdrawers”) (group × time interaction effect P = 0.040). The long-term withdrawers had better handgrip strength (P ≤ 0.006) from week 3 on than at baseline (week 0).

Among women, handgrip strength improved significantly starting from the third withdrawal week when compared to baseline values both in the short-term withdrawers (n = 45) and short-term non-withdrawers (n = 14) (*P ≤* 0.005; Table 2). Women who were benzodiazepine-free at 6 months (n = 19; “long-term withdrawers”) improved their handgrip strength more than those who were not benzodiazepine-free at 6 months (n = 40; “non-withdrawers”) (group × time interaction effect *P =* 0.040; Figure 2). In the long-term withdrawers handgrip strength was better at 3 weeks, 1 month, 2 months and 6 months compared to baseline values (*P* ≤ 0.006; Table 2).

**Table 2:** **Handgrip strength (kg) in women (** ***n =*** **59) by success of short-term (“ONE MONTH”) and long-term (“SIX MONTHS”) withdrawal**

*ONE MONTH (short-term withdrawal)*	*Measurement point*									
	*Baseline*	*1 week*	*2 weeks*	*3 weeks*	*1 month*	*2 months*	*6 months*			
	*mean,* *SD*	*mean,* *SD*	*mean,* *SD*	*mean,* *SD*	*mean,* *SD*	*mean,* *SD*	*mean,* *SD*	*P* ^*1*^	*P* ^*2*^	*P* ^*3*^
*short-term withdrawers (n = 45)*	23.5,	24.2,	24.4,	24.8*,	24.7*,	24.9*,	24.8*,	0.350	0.032	0.003
	5.4	5.8	5.5	5.6	5.7	5.5	5.8			
*short-term non-withdrawers (n = 14)*	20.4,	21.0,	19.9,	20.3,	21.0,	21.2,	20.8,			
	4.1	4.5	4.1	5.7	5.5	5.8	6.2			
***SIX MONTHS (long-term withdrawal)***	***Measurement point***									
	***Baseline***	***1 week***	***2 weeks***	***3 weeks***	***1 month***	***2 months***	***6 months***			
	***mean, SD***	***mean, SD***	***mean, SD***	***mean, SD***	***mean, SD***	***mean, SD***	***mean, SD***	***P*** ^***1***^		***P*** ^***4***^
*long-term withdrawers (n = 19)*	23.3,	24.1,	24.7,	25.6^§^,	25.2^§^,	25.6^§^,	25.3^§^,	0.040		0.001
	5.4	6.7	5.6	5.9	5.7	5.6	5.5			
*long-term non-withdrawers (n = 40)*	22.5,	23.1,	22.7,	22.9,	23.1,	23.1,	23.2,			0.427
	5.2	5.2	5.4	5.8	5.8	5.7	6.3			

*P* ^1^ = Statistical significance for group × time interaction effect; repeated measures analysis of variance.

*P* ^2^ = Statistical significance for group effect; repeated measures analysis of variance; adjusted for time.

*P* ^3^ = Statistical significance for time effect; repeated measures analysis of variance using Dunnett’s method in pairwise comparisons. After adjustment for group, handgrip strength was better* at 3 weeks (*P =* 0.002), at 1 month (*P =* 0.004), at 2 months (*P =* 0.005), and at 6 months (*P =* 0.004) compared to baseline.

*P* ^4^ = Statistical significance for time effect within groups; repeated measures analysis of variance. Handgrip strength was better^§^ at 3 weeks (*P =* 0.005), at 1 month (*P =* 0.006), at 2 months (*P <* 0.001) and at 6 months (*P =* 0.003) among withdrawers compared to baseline.

SD = Standard deviation.

### Handgrip strength in men

Among men, handgrip strength improved significantly compared to baseline values both in the short-term withdrawers (n = 24) and non-withdrawers (n = 6) (*P =* 0.002). A significant improvement in strength was seen also in those men who were benzodiazepine-free at 6 months (n = 15; long-term withdrawers) as well as in those who were not completely benzodiazepine abstinent at 6 months (n = 15; long-term non-withdrawers) (*P =* 0.002; Table 3).

**Table 3:** **Handgrip strength (kg) in men (** ***n =*** **30) by success of short-term (“ONE MONTH”) and long-term (“SIX MONTHS”) withdrawal**

*ONE MONTH (short-term withdrawal)*	*Measurement point*									
	*Baseline*	*1 week*	*2 weeks*	*3 weeks*	*1 month*	*2 months*	*6 months*			
	*mean, SD*	*mean, SD*	*mean, SD*	*mean, SD*	*mean, SD*	*mean, SD*	*mean, SD*	*P* ^*1*^	*P* ^*2*^	*P* ^*3*^
*short-term withdrawers (n = 24)*	42.9,	44.9*,	44.6*,	44.6*,	44.5*,	44.5*,	44.2*,	0.296	0.619	0.002
	6.5	5.7	6.6	5.5	6.5	5.2	6.0			
*short-term non-withdrawers (n = 6)*	40.3,	44.0*,	42.8*,	41.5*,	41.9*,	43.6*,	43.9*,			
	5.8	7.0	6.2	6.0	6.6	7.7	8.4			
***SIX MONTHS (long-term withdrawal)***	***Measurement point***									
	***Baseline***	***1 week***	***2 weeks***	***3 weeks***	***1 month***	***2 months***	***6 months***			
	***mean, SD***	***mean, SD***	***mean, SD***	***mean, SD***	***mean, SD***	***mean, SD***	***mean, SD***	***P*** ^***1***^	***P*** ^***2***^	***P*** ^***3***^
*long-term withdrawers (n = 15)*	41.5,	43.5^§^,	43.7^§^,	43.7^§^,	43.7^§^,	44.4^§^,	43.9^§^,	0.357	0.897	0.002
	5.6	5.5	5.4	5.2	6.3	4.5	5.5			
*long-term non-withdrawers (n = 15)*	43.3,	46.0^§^,	45.0^§^,	44.6^§^,	44.2^§^,	44.2^§^,	44.3^§^,			
	7.1	6.1	7.5	6.1	6.9	6.8	7.4			

*P* ^1^ = Statistical significance for group × time interaction effect; repeated measures analysis of variance.

*P* ^2^ = Statistical significance for group effect; repeated measures analysis of variance; adjusted for time.

*P* ^3^ = Statistical significance for time effect; repeated measures analysis of variance using Dunnett’s method in pairwise comparisons. After adjustment for group at one month’s time point handgrip strength was better* at 1 week (*P =* 0.002), at 2 weeks (*P =* 0.001), at 3 weeks (*P =* 0.001), at 1 month (*P =* 0.013), at 2 months (*P =* 0.001), and at 6 months (*P =* 0.006) compared to baseline. After adjustment for group at six months’ time point handgrip strength was better^§^ at 1 week (*P =* 0.002), at 2 weeks (*P =* 0.002), at 3 weeks (*P =* 0.001), at 1 month (*P =* 0.013), at 2 months (*P =* 0.001), and at 6 months (*P =* 0.006) compared to baseline.

SD = Standard deviation.

### Balance test: short Berg’s balance scale (BBS-9)

**Figure 3:**
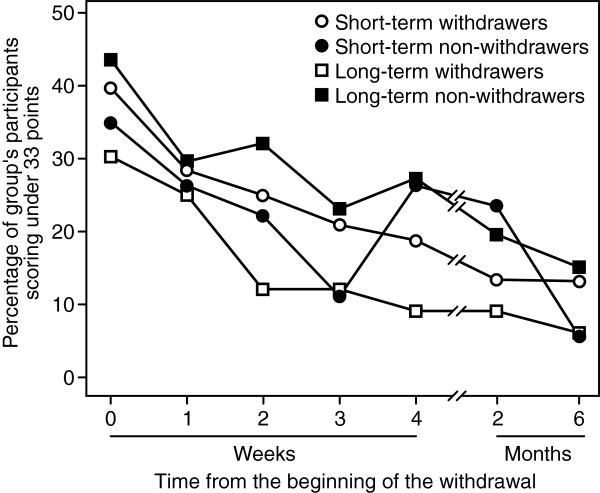
**Effect of medication withdrawal on balance.** Legend: Effect of short-term and long-term withdrawal from chronic daily use of temazepam, zopiclone and zolpidem on balance in older adults assessed using the Short Berg’s Balance Scale (BBS-9). The percentage of participants at increased fall risk (<33 points) at baseline and at different time points after the beginning of withdrawal are shown. Of note, most of the non-withdrawers also reduced their hypnotic use and were temporarily abstinent.

Balance (BBS-9 score) improved in all groups compared to baseline data (Figure 3). The improvement was significant in short-term and long-term withdrawers from the first week after initiating withdrawal (*P* ≤ 0.003; Table 4). However, the changes in BBS-9 scores did not differ between short-term withdrawers and non-withdrawers (*P =* 0.474; Table 4). Changes in BBS-9 scores reached borderline significance between the long-term withdrawers and non-withdrawers (group × time interaction effect, *P =* 0.054), but there was no difference in BBS-9 scores between groups (group effect *P =* 0.165).

**Table 4:** Balance* by success of short-term (“ONE MONTH”) and long-term (“SIX MONTHS”) withdrawal

*ONE MONTH (short-term withdrawal)*	*Measurement point*									
	*Baseline*	*1 week*	*2 weeks*	*3 weeks*	*1 month*	*2 months*	*6 months*			
	*n (%)*	*n (%)*	*n (%)*	*n (%)*	*n (%)*	*n (%)*	*n (%)*	*P* ^*1*^	*P* ^*2*^	*P* ^*3*^
*short-term withdrawers under 33 points* ^†^	27 (40)	19 (28)	17 (25)	14 (21)	13 (19)	9 (13)	9 (13)	0.474	0.997	<0.001
*short-term non-withdrawers under 33 points* ^†^	7 (35)	5 (26)	4 (22)	2 (11)	5 (26)	4 (24)	1 (6)			
***SIX MONTHS (long-term withdrawal)***	***Measurement point***									
	***Baseline***	***1 week***	***2 weeks***	***3 weeks***	***1 month***	***2 months***	***6 months***			
	***n (%)***	***n (%)***	***n (%)***	***n (%)***	***n (%)***	***n (%)***	***n (%)***	***P*** ^***1***^	***P*** ^***2***^	***P*** ^***3***^
*long-term withdrawers under 33 points* ^†^	10 (30)	8 (25)	4 (12)	4 (12)	3 (9)	3 (9)	2 (6)	0.054	0.165	<0.001
*long-term non-withdrawers under 33 points* ^†^	24 (44)	16 (30)	17 (32)	12 (23)	15 (27)	10 (20)	8 (15)			

*Using Short Berg’s Balance Scale (BBS-9).

Note: Number of withdrawers and non-withdrawers under 33 points (and their percentage from all participants) at different time points are given.

*P* ^1^ = Statistical significance for group × time interaction effect; logistic regression analysis using GEE estimation; adjusted for gender.

*P* ^2^ = Statistical significance for group effect; logistic regression analysis using GEE estimation; adjusted for gender and time.

*P* ^3^ = Statistical significance for time effect; logistic regression analysis using GEE estimation. After adjustment for gender and group at one month’s time point balance was better at 1 week (*P =* 0.003), 2 weeks (*P =* 0.003), at 3 weeks (*P <* 0.001), at 1 month (*P <* 0.001), at 2 months (*P <* 0.001), and at 6 months (*P <* 0.001) compared to baseline. After adjustment for gender and group at six months’ time point balance was better at 1 week (*P =* 0.003), at 2 weeks (*P =* 0.003), at 3 weeks (*P <* 0.001), at 1 month (*P <* 0.001), at 2 months (*P <* 0.001), and at 6 months (*P <* 0.001) compared to baseline.

^†^Cut off -point (<33) suggests increased fall risk [24].

Men and women combined (*n =* 89).

## Discussion

Our results demonstrate that withdrawal from temazepam, zopiclone and zolpidem can significantly improve handgrip strength and balance in older adults who have been long-term users of these hypnotics. The improvement starts rapidly, during the first weeks after initiating gradual benzodiazepine withdrawal. Some improvement was seen also in those participants who decreased their benzodiazepine use but did not completely discontinue it. These subjects are described here as short-term or long-term non-withdrawers, and their hypnotic use and other characteristics are described in more detail elsewhere [19, 20].

Handgrip strength is a recommended measurement to assess overall muscle strength [25]. We used the Seahan (Jamar®) handheld dynamometer to measure the patients’ maximal handgrip strength in standard testing conditions. The baseline handgrip strength results from our study are comparable with reference values from a sample of Finnish adults over 50 years of age [26]. Since there was a statistically significant gender difference concerning handgrip strength results, we analyzed men and women separately. This was necessary in order to study the true effect of drug withdrawal, but at the same time it markedly decreased sample size. This must be considered as a limitation.

Both muscle weakness and poor balance are risk factors for falls [11, 27], and benzodiazepines are known to impair muscle strength and balance [16, 28]. Even though the fundamental aim of the present study was to provide tools for fall and fracture prevention in a clinical context, this study was not designed to register participants’ falls. However, when the 89 participants were repeatedly interviewed during the 6 months, only one fracture was reported. A man had fallen from ladders when cleaning eaves and sustained a high-energy fracture of acetabulum.

Previous studies have not examined chronic users of these benzodiazepine hypnotics for a possible influence of withdrawal on muscle strength. All of our patients were relatively healthy outpatients, and, until rapid withdrawal in this study, most of them had used benzodiazepines as hypnotics daily for more than five years. Our recent study revealed that both attentional and psychomotor cognitive functioning of these same patients was impaired during benzodiazepine use at baseline when compared to a benzodiazepine-naïve control group [20]. Furthermore, cognitive impairment was still present in these subjects after 6 months of benzodiazepine abstinence suggesting a possibility of prolonged neuronal adverse effects [20]. In the present study an improvement in muscle strength and balance in the same patients could be observed starting from one to three weeks from the beginning of the withdrawal process. This finding is encouraging for clinicians working with older adults, many of whom are chronic users of benzodiazepine hypnotics.

The short version of Berg’s Balance Scale (BBS-9) was used to estimate participants’ balance. BBS-9 strongly correlates with the original BBS, and it takes a shorter time to complete [22]. Although the original BBS has been criticized for having a ceiling effect among higher-functioning older adults, BBS-9 might avoid this effect [22, 24]. It can be speculated that if the participants were more frail, the differences between withdrawers and non-withdrawers might have been detected more clearly.

A number of earlier studies have described the effects of benzodiazepine withdrawal on older participants’ health. Many of these studies have focused on the cognitive consequences of withdrawal [20, 29, 30]. However, there seems to be no previous study in elderly outpatients on the effect of withdrawal from chronic use of temazepam, zopiclone and zolpidem on handgrip strength and balance. Furthermore, studies exploring balance changes after other benzodiazepines withdrawal are rare and have limitations or methodological differences compared to our study [17, 18].

A controlled trial by Habraken and her colleagues concluded that lorazepam withdrawal improved nursing home residents’ level of daily functioning [17]. Daily functioning was assessed using observational data and no balance test or muscle strength test was performed. Their sample was limited to nursing home inpatients, which is different from our setting with relatively well-conditioned community-dwelling patients.

A recent study by Tsunoda et al. focused on the effects of benzodiazepine derivative hypnotics’ discontinuation on postural sway [18]. The 26 participants in the study were living in nursing homes and had psychiatric diagnoses – mainly schizophrenia and dementia. Balance was assessed using the Clinical Stabilometric Platform (ANIMA® GS-7, Tokyo). After discontinuation of hypnotic use, the range and total trunk motion length were significantly shortened, i.e., balance was improved. However, the characteristics of that study’s participants differed from those in our study, as their participants had psychiatric diagnoses and were significantly older (mean age ± SD was 79.1 ± 8.9 years) than our participants (66.7 ± 6.9 years). The older and cognitively diminished (Mini Mental State Examination mean 21.6, SD ±4.8) participants in Tsunoda’s study were more vulnerable and seem to have benefited even more from benzodiazepine withdrawal than our participants did.

Our study has strengths and limitations. First, the number of participants (altogether 89) is larger than in other studies that have examined the effect of withdrawal from chronic use of benzodiazepines on physical performance [17, 18]. However, our study power did not allow analyzing the effects of temazepam, zopiclone or zolpidem separately. Second, we confirmed the use of benzodiazepines at baseline and at the end of the one-month withdrawal period by measuring plasma concentrations of individual benzodiazepines. During the follow-up period, up to six months, information concerning benzodiazepine use was collected by interviews and by checking each patient’s medical records and prescription history. These methods are reliable, but using different data collection methods may raise questions about comparability between the results from blood samples vs those from interviews or prescription records. Third, all of our participants were long-term users of benzodiazepine hypnotics who voluntarily participated in and were well motivated to complete the benzodiazepine withdrawal study. However, the absence of an initially benzodiazepine naïve, “pure” control group leaves open the issue of how a benzodiazepine-free population would have performed in the various tests. Finally, non-withdrawers also markedly reduced their benzodiazepine use during the study [19]. This reduction in benzodiazepine use most likely explains their enhanced handgrip strength and balance results. The difference between the results of the withdrawers and non-withdrawers (more correctly “partial withdrawers”) could have been clearer if the non-withdrawers had not reduced their benzodiazepine doses. On the other hand, it is encouraging that reduction of benzodiazepine use, and not only their complete withdrawal, can result in improved muscle strength and balance in chronic users.

A significant percentage of elderly patients regularly use benzodiazepines or related drugs [3, 31, 32], and the adverse effects of chronic benzodiazepine use on attentional and psychomotor cognitive functioning can persist for several months after the withdrawal [20] *.* The present study demonstrates that beneficial effects of benzodiazepine withdrawal on muscle strength and balance can be seen within one month after initiating withdrawal. This is clinically important as impaired muscle strength and balance are risk factors for falls.

The present study and previous studies from the same participants show that the laborious withdrawal process produces good withdrawal outcomes, but some participants still remain or return regular users of benzodiazepines. Thus, it seems better to avoid prescribing them at all; that is “prevention is better that cure”. Regardless, our results encourage discontinuing prolonged use of benzodiazepine hypnotics.

## Conclusions

An improvement in handgrip strength and balance was observed in older outpatients after temazepam, zopiclone and zolpidem withdrawal. These positive effects encourage discontinuing long-term use of benzodiazepine-type hypnotics, particularly in older women who are at a high risk of sustaining fall and fractures.

## Authors’ information

JN: MD, PhD, Clinical Teacher (General Practice); JP: MD, PhD, Specialist in Neurology; RL: MD, Specialist in General Practice; TV: MSc, Biostatistician; AL: MPH, ScD, Professor; MP: MD, PhD, Specialist in Neurology, Professor; IR: MD, PhD, Specialist in Internal Medicine and Geriatrics, Emeritus Professor; PJN: MD, PhD, Specialist in Clinical Pharmacology, Emeritus Professor; SLK: MD, PhD, Specialist in General Practice and Geriatrics, Emerita Professor.
